# Psychic Effects of the San Francisco Earthquake

**Published:** 1906-08-04

**Authors:** 


### Psychic Effects of the San Francisco Earthquake.

The current number of the *Pacific Medical Journal* contains an interesting account of the San Francisco earthquake. The entire plant of the *Journal* was destroyed by the fire which followed the earthquake. Not a single volume of the library was saved, and the editor asks for a donation of any back numbers of the *Journal,* as there are none left. Mr. H. D'Arcy Power writes to the *Journal* the following acount of the psychic effects of the earthquake. He says that when the earthquake occurred half a million people were in bed and the functional activity of their nervous systems had just been recuperated and brought up to the normal physiological standard by the night's rest. The earthquake, without a moment's warning, placed this half million face to face with death, and the majority realised the nature of the peril. Yet there was no exhibition of fear or despair. "I was in the storm-centre of the earthquake—namely, in the immediate vicinity of the City Hall—and I am prepared to assert that notwithstanding the universal ruin that befell the brick buildings so plentifully surrounding that structure, there were not more than fifty people in the street five minutes after the earthquake had ceased, that only very few came out minus clothing, and that none showed abject fear. I stood between the ruined Supreme Court building which has more than two hundred rooms, the Strathmore with more than a hundred, and the Argyll containing still more. The inhabitants had been subjected to the terrifying experience of these large brick buildings falling in large part into the street accompanied by the thunder of the collapse of the City Hall, and yet they stayed to dress before quitting their shattered rooms. The population is mixed in race in this district, but I noticed no difference to speak of in the behaviour of its different elements." The trial came at a time when the nervous system was well rested by sleep; it might have been otherwise had it come at the end of a hot and harrowing day. The fire followed and for three days the mass of the people were subjected to a strain such as no similar body of our fellow creatures has endured in this generation. At the end of that time between two and three hundred thousand people were homeless, destitute, and for the most part had to start life over again. They had suffered loss of sleep, many were hungry, and all had the prospect of immediate famine ahead. By all the precedents of history these hundreds of thousands should have been in the slough of despair. Men should have slunk along with white, despairing faces; women should have wept, and children wailed. Nothing could have been further from the truth. Some men looked worried and depressed; most seemed to be in excellent good humour with themselves and the world in the knowledge that they and theirs were still living. In a walk of ten miles no woman was seen crying. The mental condition was rather one of mild excitement. A few observations on the pulse-rate showed an acceleration of ten to twenty beats a minute. The explanation seems to be that nearly everyone had been in personal danger and the danger was passed and the long tension relieved. By the enforced recourse to a few open encampments men were thrown together, and each time friend met friend the sense of joyous relief was quickened. The green of the fields and the blue of the sky aided the reaction, for it was lovely weather. Finally, the worn-out bodies and overwrought minds of the masses made them an easy prey to the power of suggestion and they felt the comfort of rest after exceeding weariness. Had the weather been bad or the preliminary strain less, the power of suggestion might have worked in the opposite direction, and a despairing multitude might well have replaced our good-natured and hopeful crowds. There was, too, an improvement in the general health of the people after the earthquake. It is an undoubted fact that a great many men and women who were in a poor state of health before the shock with bad appetites and defective digestion are now eating all they can get and digesting it without trouble; whilst the mental condition which so often accompanies the dyspeptic state has equally improved. The explanation is as simple as it is rational. These people were fortunately deprived of their trams, alcohol, and luxuries; they had nothing but simple food, and they were compelled to take exercise in the open air to get it. The men have found it possible to live without cigars or whisky, and the ladies without candy. They have cooked their simple meals in the streets to the better ventilation of their houses; for lack of light they have gone to bed early with the compensation that they have risen with the lark. They have had the enforced benefits of a sanatorium, an[illegible] [illegible]ood health is the result.

